# Long-Term Results of a Prospective Multicenter Trial of APBI with Photon IORT

**DOI:** 10.3390/cancers17111762

**Published:** 2025-05-23

**Authors:** Laura García-Cabrera, Beatriz Pinar-Sedeño, María Auxiliadora Cabezón-Pons, Nieves Rodriguez-Ibarria, Alba Dominguez-Dominguez, Daniel Aguiar-Santana, Paula Martín-Barrientos, Irene Rey-López, Pedro C. Lara, Marta Lloret-Saez-Bravo

**Affiliations:** 1 Radiation Oncology Department, Dr. Negrin University Hospital Las Palmas GC, 35010 Las Palmas de Gran Canaria, Spain; lgarcabn@gobiernodecanarias.org (L.G.-C.); mpinsed@gobiernodecanarias.org (B.P.-S.); mcabpon@gobiernodecanarias.org (M.A.C.-P.); nrodiba@gobiernodecanarias.org (N.R.-I.); adomdomt@gobiernodecanarias.org (A.D.-D.); dagusan@gobiernodecanarias.org (D.A.-S.); pmarbar@gobiernodecanarias.org (P.M.-B.); ireylop@gobiernodecanarias.org (I.R.-L.); 2 Medical School, Las Palmas University, 35001 Las Palmas de Gran Canaria, Spain; 3 Canarian Insitute for Cancer Research, 38204 San Cristobal de La Laguna, Spain; 4 Oncology Department, Canarian Comprehensive Cancer Center, San Roque University Hospital, 35001 Las Palmas de Gran Canaria, Spain; 5 Department of Medicine, Fernando Pessoa Canarias University, 35001 Las Palmas de Gran Canaria, Spain

**Keywords:** IORT, Intrabeam, APBI, breast, exclusive

## Abstract

Purpose: The aim of the present study is to analyze, for the first time, the results of a large prospective academic multicenter trial of partial breast irradiation (PBI) with exclusive photon intraoperative radiation therapy (ph-IORT) in early breast cancer patients, focusing on ipsilateral breast tumor recurrence. The secondary endpoints were (a) incidence of regional/distant recurrence, (b) survival, and (c) toxicity. Methods: From January 2013 to December 2022, patients with low-risk TARGIT-A criteria invasive breast cancer were included in a prospective academic multicenter study of exclusive PBI with ph-IORT during breast-conserving surgery, conducted in three university hospitals in Las Palmas (Hospital Universitario Insular, Hospital Universitario Materno-Infantil, and Hospital Universitario de Gran Canaria Dr. Negrín). Results: Three hundred and twelve patients were included in the study. The mean age at diagnosis was 62 years (46–88). All tumors were classified as luminal molecular profile. No patient received supplementary external beam radiotherapy. Four patients developed ipsilateral breast tumor recurrence (IBTR) at 19, 29, 43, and 62 months of follow-up. Seventeen patients died of intercurrent diseases, and there were only 2 breast cancer-related deaths at 68 and 95 months, respectively. With a median follow-up of 78 months (7–140), actuarial 5-year freedom from local relapse and cancer survival rates were 98.9% and 100%, respectively. No patient developed early or late grade-3 toxicity. Conclusions: Partial breast irradiation with ph-IORT is a feasible, safe, and useful treatment in early breast cancer patients after BCS. A longer follow-up is needed to confirm the present results.

## Simple Summary

The aim of the present study is to analyze, for the first time, the results of a large prospective academic multicenter trial of partial breast irradiation (PBI) with exclusive photon intraoperative radiation therapy (ph-IORT) in early breast cancer patients, focusing on ipsilateral breast tumor recurrence. From January 2013 to December 2022, 312 patients with low-risk invasive breast cancer were included in a prospective academic multicenter study of exclusive PBI with ph-IORT during breast-conserving surgery, conducted in three university hospitals in Las Palmas. Four patients developed ipsilateral breast tumor recurrence (IBTR) at 19, 29, 43, and 62 months of follow-up. Actuarial 5-year freedom from local relapse and cancer survival rates were 98.9% and 100%, respectively. No patient developed early or late grade-3 toxicity. PBI with ph-IORT is a feasible, safe, and useful treatment in early breast cancer patients after BCS. A longer follow-up is needed to confirm the current results.

## 1. Introduction

Whole-breast irradiation (WBI) has been shown to increase local control and survival after breast-conserving surgery (BCS) [1]. Most recurrences after conservative surgery (ipsilateral breast tumor recurrence (IBTR)) occur in the vicinity of the tumor bed [2]. This fact raised the possibility of using partial breast irradiation (PBI) after conservative surgery for early-stage breast cancer [2].

Large randomized trials of partial breast irradiation using external beam radiotherapy (EBRT) [3,4] or brachytherapy [5] have shown ipsilateral breast tumor recurrence rates comparable to whole-breast irradiation and are widely included in international guidelines [6,7]. Randomized trials using intraoperative radiotherapy (IORT) have shown either negative [8] or controversial [9] results. Patient selection characteristics [10] or the delayed use of IORT [11,12] in a second surgery could explain these results.

In the photon IORT Targeted Intraoperative A (TARGIT A) trial, a prespecified sub-analysis of patients treated with IORT at the time of surgery showed non-inferior local control rates compared to whole-breast irradiation [13]. Long-term follow-up results of IORT administered during surgery were deemed non-inferior compared to whole-breast irradiation (5-year IBRT = 2.11% IORT vs. 0.95% WBI, 95% CI = 0.32–1.99%), without any difference in survival [13]. The use of adjuvant “adapted” whole-breast radiotherapy in 21% of the IORT arm within this subgroup (pre-pathology stratum) has been suggested as a potential source of bias when assessing the role of photon IORT as partial breast irradiation therapy in early breast cancer [14].

Interestingly, there were no differences in breast cancer mortality rates when comparing the IORT and the whole-breast irradiation groups in these two randomized IORT trials [8,9]. Furthermore, the non-breast cancer mortality was significantly reduced (*p* = 0.005) in the TARGIT A trial [13]. A meta-analysis of randomized trials comparing partial breast irradiation with whole-breast irradiation showed a significant reduction in non-breast cancer deaths among patients treated with partial breast irradiation [15].

The aim of the present study is to analyze, for the first time, the results of a large prospective academic multicenter trial of PBI with exclusive photon IORT (ph-IORT) in early breast cancer patients, with the incidence of ipsilateral breast tumor recurrence (IBTR) being the primary objective. The secondary endpoints of the present study were (a) regional relapse, (b) distant metastasis, (c) survival (cause-specific and overall survival), and (d) toxicity.

## 2. Methods

### 2.1. Study Design and Participants

This multicenter prospective study was conducted in three academic university hospitals in the Las Palmas province (Hospital Universitario Materno Infantil, Hospital Universitario Insular, and Hospital Universitario General de Gran Canaria Dr. Negrín) from January 2013 to December 2022. Ethics Committee approval and written informed consent were obtained from all patients. The patients were followed prospectively according to Spanish regulation RD 1566/1998 for the Quality Assurance of Radiotherapy [16].

Women referred to the academic hospitals in the Las Palmas province with suspicious breast cancer were routinely studied using mammography and ultrasonography. Breast magnetic resonance imaging (MRI) or subtraction mammography were also performed, if necessary. Cancer histology was confirmed using core-needle biopsy. Cancer staging was performed according to the 8th edition of the Tumor, Node, Metastasis (TNM) classification system [17]. All cases were discussed by the multidisciplinary tumor boards (MTBs) at the respective hospitals.

Among all patients diagnosed with breast cancer in the three participating academic hospitals of the Las Palmas province, those fulfilling the following inclusion criteria were considered candidates for exclusive partial breast irradiation with photon IORT: (a) age over 45 years, (b) no preoperative systemic chemotherapy administered, (c) invasive carcinoma of any grade, (d) unifocal tumor with a diameter ≤ 3 cm, (e) cN0, (f) M0, (g) no lobular histology, and (f) luminal molecular profile (positive estrogen receptor with any progesterone receptor, Her2 expression, or Ki67 percentage). A Ki67 percentage over 15% was considered positive. Her2 expression was studied using immunohistochemistry. Tumors with IHC reports 2+ or 3+ were considered for fluorescence in situ hybridization (FISH) analysis. Final Her2 expression in these cases was scored according to the FISH results.

All patients were treated with breast-conserving surgery and sentinel node biopsy. Surgical margin status was evaluated intraoperatively, and macroscopic tissue negative margins were required for immediate ph-IORT at the time of lumpectomy. Photon IORT treatment at the participating institutions was delivered through a low-energy (50 KV) portable X-ray accelerator, Intrabeam^®^ (Carl-Zeiss, Ober Kochen, Germany), with the prescribed radiation dose of 20 Gy to the applicator surface.

After surgery, all patients were re-discussed at the multidisciplinary tumor board of each participating center. After a definitive pathology report, exclusion criteria included risk factors recommending complementary external radiotherapy, such as surgical margin < 1 mm, any resection after IORT, lymphovascular invasion, lobular histology, extensive ductal in situ component, and nodal involvement (pN1).

Adjuvant systemic treatment was decided by the MTB of every participating hospital according to state-of-the-art National Comprehensive Cancer Network (NCCN) guidelines [18]. All patients were prescribed hormonal treatment. Patients with Ki67 > 15% were referred for adjuvant systemic chemotherapy according to genomic platform risk scores (when needed), comorbidities, and the patient’s acceptance of chemotherapy. Patients with Her2 overexpression were referred for systemic adjuvant chemotherapy and anti-Her2 treatment.

### 2.2. Follow-Up

All patients underwent clinical examination at 1, 3, and 6 months after photon IORT, then every 3 months until the 2nd year of follow-up, every six months until the 5th year, and annually thereafter. Breast mammograms and ultrasonography were routinely performed once a year. MRI, computerized tomography (CT), bone scans, and positron emitting tomography (PET)-CT were performed only when relapse was suspected. Follow-up was performed jointly by the treating physicians from the participating institutions. Late adverse effects were scored using the Common Terminology Criteria for Adverse Events (CTCAE) 5.0 scale [19]. No patient was lost to follow-up during the study.

### 2.3. Outcomes

Local and regional relapse were defined as reappearance of the tumor in the ipsilateral breast and in the ipsilateral nodes, respectively. The presence of metastases outside the breast or the ipsilateral regional nodes was considered as a distant recurrence. The time to recurrence was the interval between surgery and diagnosis of relapse. Survival status and length of follow-up were calculated until the date of the patients’ last follow-up visit.

Ipsilateral breast tumor relapse rates were the primary objective of the present study. Secondary endpoints include (a) regional relapse rates, (b) distant metastasis, (c) survival (cause-specific/overall survival), and (d) toxicity.

### 2.4. Statistical Analysis

Time-to-event curves were estimated using the Kaplan–Meier method and compared using a two-sided log-rank test. A probability level of 0.05 was considered statistically significant. Statistical analyses were performed using SPSS version 26 (IBM Corp., Armonk, NY, USA).

## 3. Results

### 3.1. Patients and Treatment

**Figure 1:**
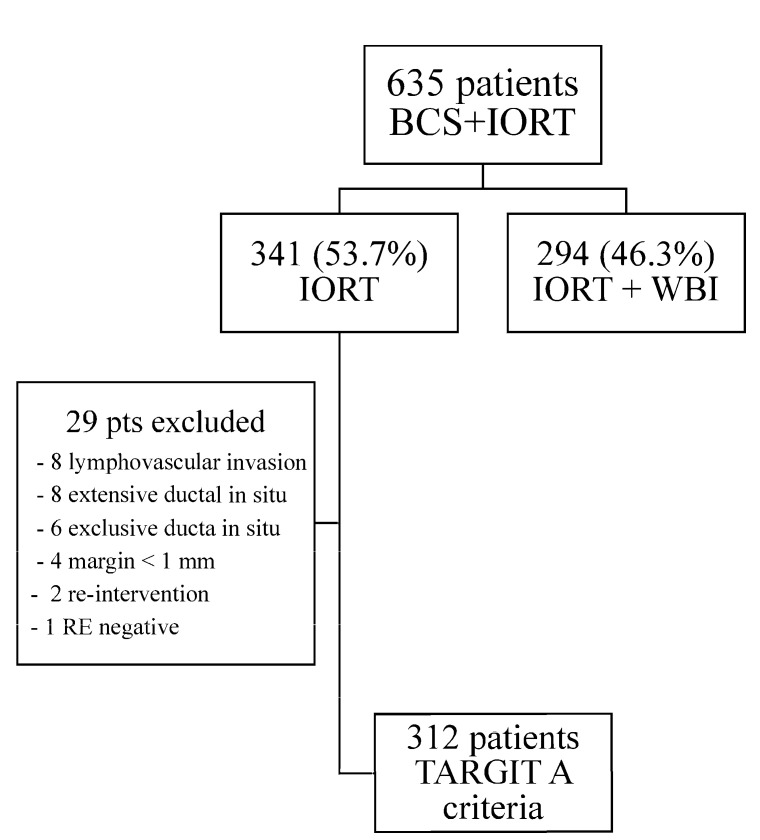
Patient’s Flow Chart.

Between January 2013 and December 2022, 653 patients fulfilling the inclusion criteria for photon IORT treatment at the time of breast-conserving surgery were included in the study. After the final pathology report, 294 (46.3%) patients showed at least one of the exclusion criteria defined in our protocol. Therefore, 341 patients were treated using exclusive partial breast irradiation with photon IORT at the three participating academic centers. A monitoring review of the trial showed that 29 patients (out of 341) had to excluded from the study, as they met the criteria for adapted external beam whole-breast irradiation (lymphovascular invasion in 8 cases, extensive ductal in situ in 8 cases, ductal in situ carcinoma in 6 patients, margins < 1 mm in 4 cases, second surgery in 2 cases, and 1 non-luminal patient). Finally, 312 patients who fulfilled the TARGIT A criteria for exclusive PBI with photon IORT were eligible for analysis. Figure 1 describes the patients’ selection flowchart.

The patients’ tumor characteristics and treatments are summarized in Table 1. The patients’ mean age was 62 years (range 46–88), and 276 out of 312 (88.5%) were postmenopausal. Only 11 cases were classified as T2 (3.2%), and 304 out of 312 cases (97.4%) were grade 1–2 tumors. All 312 patients had ER+ tumors and 280 (89.7%) had PR+ tumors. Ki67% was positive in 85 patients, and 93 out of 312 patients were luminal B (10 patients also were Her-2 positive).

Photon IORT at the time of breast-conserving surgery was successfully delivered in all cases. A dose of 20 Gy was prescribed to the applicator surface. The median applicator size used was 4 cm (range: 2–5).

All but 15 (297/312) very low-risk patients (95.2%) received adjuvant systemic hormonal treatment according to menopausal status. Forty-four patients also received adjuvant chemotherapy due to high Ki67% and/or Her2 overexpression. The patients’ age, comorbidities, and acceptance were important factors when administering adjuvant chemotherapy in luminal cases. Eight out of ten Her2+ patients received the proposed anti-Her2 treatment. In 2 patients bearing Her2 overexpressing tumors, Her2 treatment was not administered due to age and comorbidities. The follow-up was closed in June 2024.

**Figure 2:**
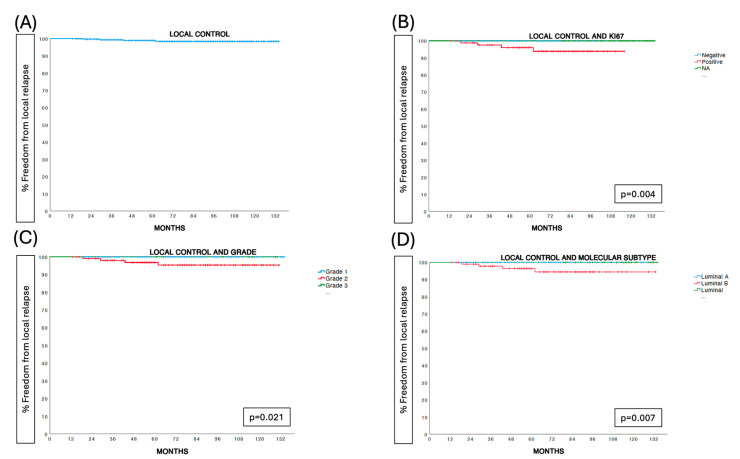
Prognostic factors for local control.

After a median follow-up of 78 months (range 7–140), there were 4 ipsilateral breast tumor recurrences at 19, 29, 43, and 62 months, respectively. In total, 19 women died during follow-up: 2 due to breast cancer-related causes at 68 and 95 months and 17 due to non-breast cancer-related causes. The five-year estimated ipsilateral breast tumor recurrence risk was 1.1% (95% confidence interval (CI) = 0.4–1.8%). Molecular luminal B subtype (*p* = 0.007), positive Ki67 status (*p* = 0.004), and tumor grade 2 (*p* = 0.021) were predictive factors for local relapse (Table 2) (Figure 2).

The in-breast recurrence analysis showed that 3 out of 4 recurrences were in the tumor bed and 1 out of 4 was elsewhere in the breast. The median time between surgery and local recurrence was 38.25 months, with a range of 19–62. Two patients received adjuvant endocrine therapy, one patient referred for endocrine therapy refused treatment, and one patient received adjuvant chemotherapy followed by endocrine therapy at the time of the primary treatment. The tumor histology at recurrence was ductal infiltrating carcinoma in 3 cases and ductal carcinoma in situ in 1 patient. Treatment of the 4 local relapses included mastectomy in 3 patients and a new breast-conserving surgery followed by whole-breast irradiation in 1 patient. Five-year mastectomy-free survival was 98.9%. No regional relapses were observed. The five-year estimated freedom from regional relapse rate was 100%. Distant metastases appeared in 3 out of 312 patients, resulting in a 5-year freedom from distant relapse rate of 99.6% (95% CI = 96.7–99.3%). Adjuvant systemic treatment in these three cases was as follows: none in the patient (described above) who refused treatment, and chemotherapy and endocrine therapy in the other two cases.

Five-year breast cancer-specific and overall survival rates were 100% and 96.8% (95% CI = 95.7–97.9%), respectively (Figure 2).

We performed a Kaplan–Meier analysis on 209 out of the 312 patients treated before June 2019, who had a minimum follow-up of 5 years. The Kaplan–Meier estimate of the 5-year IBTR for these patients was 1.4%.

### 3.2. Toxicity

Immediate post-surgical toxicity observed included seroma drained > 3 times in 4 cases (1.3%), delayed wound healing in 11 cases (3.5%), and infection requiring antibiotics or surgery in 1 patient (0.3%). No hematoma draining was needed. Late grade 2 hyperpigmentation was observed in 3 out of 312 patients (1%) and grade 2 induration in 73 out of 312 patients (23.4%). No patient showed severe grade 3 late toxicity.

## 4. Discussion

The role of photon intraoperative radiotherapy as a form of partial breast irradiation has been a controversial issue over the last decade [20,21,22]. The inclusion of patients who received intraoperative radiotherapy in a delayed second surgery (post-pathology stratum) and the use of whole-breast irradiation in approximately 21% of the IORT arm cases (who received adapted whole-breast irradiation) raised concerns about fully accepting photon IORT as standard partial breast irradiation [14].

In our multicenter prospective study, only patients receiving IORT at the time of breast-conserving surgery and without any “adapted” complementary whole-breast irradiation were included. Data on local 5-year ipsilateral breast tumor recurrence (1.1%) are comparable to those published in the partial breast irradiation arms of the Florencia trial using external beam radiotherapy (1.5%, 95% CI = 0.1–3.0%), the Groupe Européen de Curiethérapie and the European SocieTy for Radiotherapy & Oncology (GEC-ESTRO) brachytherapy trial (1.44%, 95% CI: 0.51–2.38%), and the IMPORT-LOW trial (0.5%, 95% CI: 0.2–1.4%). The patients’ inclusion criteria were according to the GEC-ESTRO classification of low-risk characteristics of ipsilateral breast tumor recurrence.

Unfortunately, very few studies have been published analyzing the role of exclusive photon IORT with Intrabeam as PBI in early breast cancer [23]. Also, few studies have conducted sub-analyses of patients treated exclusively with IORT within their published series [24,25,26,27] (Table 3). All these studies were either retrospective or from single treating centers. Our large prospective multicenter trial offers, for the first time, excellent results in terms of local control, and other tumor-related outcomes without severe late toxicity. Our results are comparable with those obtained using EBRT/brachytherapy PBI [3,4,5] and shed light on the role of photon IORT in selected early breast cancer patients.

Our results could be explained by some factors that provide context for the findings and highlight their potential reproducibility:

(a) Strict adherence to patient selection characteristics outlined by the TARGIT A criteria for exclusive IORT, which also align with the GEC-ESTRO low-risk criteria (good candidates) for partial breast irradiation, was maintained. Our inclusion criteria followed those proposed in the TARGIT A trial: age over 45 years, no preoperative systemic therapy administered, invasive carcinoma of any grade, unifocal tumor with a diameter ≤ 3 cm, cN0, M0, and no lobular histology. Patients with risk factors necessitating complementary external radiotherapy, such as surgical margin < 1 mm, any resection after IORT, lymphovascular invasion, lobular histology, extensive ductal in situ component, and nodal involvement (pN1), were excluded from the study.
(b) We also included only luminal cases. Luminal B (Her2+ and highly proliferating tumors) were also included if they were not considered candidates for primary systemic therapy. Patients with high-risk molecular factors (Her2+/triple negative) and/or advanced tumors (node positive, T > 3 cm) were referred for primary systemic therapy (if necessary).
(c) Our study was carried out in 3 academic hospitals in the Las Palmas province with immediate access to conservative surgery and intraoperative radiotherapy. Furthermore, after the TARGIT A publications, it was shown that the post-pathology cohort had poorer results for local control within the breast compared to those receiving immediate intraoperative radiotherapy. Therefore, no post-pathology patients were included in our study.
(d) As per protocol, our patients were not candidates for adjuvant WBI according to the TARGIT A guidelines. Furthermore, our patients were GEC-ESTRO candidates for partial breast irradiation, so no indication for whole-breast irradiation was acknowledged.
(e) All patients in our study had luminal profiles; therefore, all of them were recommended to receive hormonal adjuvant treatment, either alone or in combination with chemotherapy or anti-Her2treatment according to guidelines. Unfortunately, 15 cases (4.8%) had medical contraindications or refused hormonal treatment.

Another novel aspect of our study is the role of tumor proliferation, as estimated by Ki67, in the prediction of ipsilateral breast tumor recurrence in these patients. In fact, all local relapses were observed in Ki67-positive tumors (4 out of 85 patients) compared to none in the 227 patients with negative or unknown Ki67 tumors (*p* = 0.004). Grade 2 tumors were also related to significantly increased IBRT rates (*p* = 0.021). To date, no published data are available regarding the role of Ki67 expression in patients treated with photon IORT. To our knowledge, the predictive value of the Ki67 tumor proliferation index for local relapse has been only demonstrated in intraoperative radiotherapy with electrons (IOERT) [28]. A multicenter prospective study of partial breast irradiation with brachytherapy found similar results [29]. As conducting a multivariate Cox regression model is not feasible due to the limited number of events, we should consider the exploratory, non-confirmatory nature of these findings. Tumor proliferation should be further studied in other trials to better define its role in selecting appropriate candidates for partial breast irradiation treatment.

Photon IORT, in our study and in others [23,27], demonstrated an excellent toxicity profile (0% grade 3 late toxicity in our series). The reduced dose for other dose-limiting tissues (heart and lung) could be the reason for the reduced non-breast cancer-related death rates observed in photon IORT [9] and partial breast irradiation studies [15]. Furthermore, the combination of photon IORT and hormonotherapy in our low-risk breast cancer patients obtained excellent local control and survival rates. Therefore, it could be suggested that elderly, low-risk breast cancer patients would be good candidates for treatment de-escalation protocols. Unfortunately, most de-escalating trials have been designed to evaluate the suppression of WBI in patients treated with endocrine therapy (ET) [30]. In a recently published meta-analysis of such prospective randomized trials, a higher rate of local relapses without an impact on breast cancer survival was observed. Endocrine therapy was also associated with limiting toxicity and a reduced quality of life [31,32].

Partial breast irradiation (with photon IORT) could represent an excellent form of radiotherapy for low-risk patients. Trials of partial breast irradiation vs. endocrine therapy assessing not only clinical outcomes but also quality of life are already ongoing [33,34]. A pre-planned interim analysis of the EUROPA trial at 24 months demonstrated a better quality of life in patients treated with partial breast irradiation compared to those receiving endocrine therapy, opening the future possibility of expanding the role of partial breast irradiation to elderly low-risk breast cancer patients [34].

The limitations of our study include a short follow-up period (78 months) and a small number of patients. Additional limitations that should be considered include the following: (1) less than a 5-year follow-up for one-third of the patients included in the 5-year ipsilateral breast tumor recurrence analysis; (2) the inability to perform multivariate analysis, fortunately due to a low event count; and (3) the lack of patient-reported outcomes, such as quality of life or satisfaction, which are critical in de-escalation trials.

## 5. Conclusions

Our results confirm, for the first time in a multicenter prospective study, that exclusive photon IORT provides local control and survival rates with excellent toxicity profiles that are comparable to other PBI techniques for low-risk early breast cancer patients. A longer follow-up is needed to confirm these results.

## Figures and Tables

**Table 1:** Patient´s characteristics.

	Patients
**Age (years)** Mean 62 (46–88)	
<70	257 (82.4%)
≥70	55 (17.6%)
**Menopausal status**	
Premenopausal	36 (11.5%)
Postmenopausal	276 (88.5%)
**T stage**	
pT1mi	1 (0.3%)
pT1a	10 (3.2%)
pT1b	143 (45.8%)
pT1c	147 (47.2%)
pT2	11 (3.5%)
**Grade**	
1	200 (64.1%)
2	104 (33.3%)
3	8 (2.6%)
**ER status**	
Positive	312 (100%)
**PR status**	
Positive	280 (89.7%)
Negative	32 (10.3%)
**Ki67 status**	
Positive	85 (27.2%)
Negative	180 (57.7%)
Unknown	47 (15.1%)
**Her2 status**	
Positive	10 (3.2%)
Negative	301 (96.5%)
Unknown	1 (0.3%)
**Molecular subtype**	
Luminal A	183 (58.7%)
Luminal B	93 (29.8%)
Luminal	36 (11.5%)
**Chemotherapy**	
Yes	44 (14.1%)
No	268 (85.9%)
**Endocrine therapy**	
Yes	297 (95.2%)
No	15 (4.8%)
**Anti HER2 therapy**	
Yes	8 (2.6%)
No	304 (97.4%)

**Table 2:** Predicting factor of local control. Patients’ tumor and postoperative-treatment characteristics.

	Patients	5 y IBTR (95%CI)	*p* Value
**Age at diagnosis**			
<70 y	257 (82.4%)	0.5% (0–1%)	0.084
≥70 y	55 (17.6%)	3.9% (1.2–6.6%)	
**Menopausal status**			
Premenopausal	36 (11.5%)	0%	0.425
Postmenopausal	276 (88.5%)	1.2% (0.5–1.9%)	
**Surgical margins distance**			
≤1 mm	33 (10.6%)	3.1% (0–6.2%)	0.667
>1 & ≤10 mm	202 (64.7%)	0.5% (0–1%)	
>10 mm	77 (24.7%)	2.2% (0–4.4%)	
**Tumor status**			
pT1	301 (96.5%)	1.1% (0.4–1.8%)	0.705
pT2	11 (3.5%)	0%	
**Tumor grade**			
1	200 (64.1%)	0%	0.021
2	104 (33.3%)	3.2% (1.4–5%)	
3	8 (2.6%)	0%	
**PR status**			
Positive	280 (89.7%)	0.8% (0.3–1.3%)	0.279
Negative	32 (10.3%)	4.3% (0–8.6%)	
**Her2 status**			
Positive	10 (3.2%)	0%	0.924
Negative	301 (96.5%)	1.1% (0.4–1.8%)	
**Ki67 status**			
Positive	85 (57.7%)	4% (1.7–6.3%)	0.004
Negative	180 (57.7%)	0%	
Unknown	47 (15.1%)	0%	
**ESTRO risk**			
Low	253 (81.1%)	0.5% (0–1%)	0.147
Intermediate	59 (18.9%)	3.5% (1–6%)	

**Table 3:** Published studies of IBTR rates at 5 y in (A) randomized PBI trials and (B) exclusive phIORT series.

(A) PBI vs. WBI Studies	Type of Study	Centers	PBI Patients	PBI Technique	IBTR (5 y)
FLORENCE (2015) [3]	Randomized	Multicenter	260	EBRT	1.5%
IMPORT LOW (2017) [4]	Randomized	Multicenter	669	EBRT	0.5%
GEC-ESTRO (2016) [5]	Randomized	Multicenter	633	Brachy	1.44%
ELLIOT (2013) [8]	Randomized	Multicenter	651	IOERT	4,2%
TARGIT A (2014) [9]	Randomized	Multicenter	1721 *	Ph-IORT	3.3%
(B) Exclusive phIORT	Type of study	Centers	PBI Patients	PBI technique	IBTR (5 y)
Tallet A (2020) [24]	Retrospective	Multicenter	406 #	phIORT	1.5%
TARGIT R(2021) [25]	Retrospective	Multicenter	477 #	phIORT	8%
Guillerm (2022) [26]	Retrospective	Unicenter	191 #	phIORT	1.7%
Laplana M, (2022) [23]	Prospective	Unicenter	50	phIORT	2.9%
Vinante et al (2024) [27]	Prospective	Unicenter	491 #	phIORT	2,5%
Present study (2025)	Prospective	Multicenter	312	phIORT	1,1%

* 21.2% received adjuvant WBI. # Subgroup analysis of a published series of patients.

## Data Availability

The data presented in this study are available on request from the corresponding author.
